# Supplementary material for: Future Sequon Finder - A novel approach for predicting future N-linked glycosylation sequon locations on viral surface proteins
Source: PLoS One. 2025 Aug 14;20(8):e0328174. doi: 10.1371/journal.pone.0328174 (PMC12352829; doi:10.1371/journal.pone.0328174)
Supplement: Attachment — Submitted filename: Response.pdf [file pone.0328174.s005.pdf]

SCHOOL OF MEDICINE & DENTISTRY

Clinical and Translational Science Institute

UNIVERSITY of ROCHESTER MEDICAL CENTER

Wednesday, June 25, 2025

Dear Editor:

Please find attached our response to reviewers for our **revised** manuscript entitled “Future Sequon Finder - A novel approach for predicting future N-linked glycosylation sequon locations on viral surface proteins” submitted for potential publication as a *research article*.

We thank the reviewers for their comments, and include our response below.

1. **Please ensure that your manuscript meets PLOS ONE's style requirements, including those for file naming.**

   We have changed the file names of our supplemental figures and we have updated the formatting of the author affiliations to be in accordance with PLOS One’s formatting guidelines.

2. **Please note that PLOS ONE has specific guidelines on code sharing for submissions in which author-generated code underpins the findings in the manuscript. In these cases, we expect all author-generated code to be made available without restrictions upon publication of the work. Please review our guidelines at https://journals.plos.org/plosone/s/materials-and-software-sharing#loc-sharing-code and ensure that your code is shared in a way that follows best practice and facilitates reproducibility and reuse.**

   In accordance with PLOS ONE's guidelines, we have created a publicly accessible GitHub repository containing all author-generated code used in this study. To ensure long-term accessibility, we have archived the repository with Zenodo and generated a DOI. The DOI has been cited in the manuscript.

3. **Please state what role the funders took in the study. If the funders had no role, please state: "The funders had no role in study design, data collection and analysis, decision to publish, or preparation of the manuscript."**

   We have amended the statement.

4. **Please include captions for your Supporting Information files at the end of your manuscript, and update any in-text citations to match accordingly.**

   The captions for our Supporting Information files have been adjusted to match PLOS One’s formatting requirements. Optional figure legends have been omitted due to the self-explanatory nature of the file names.

5. **Please review your reference list to ensure that it is complete and correct. If you have cited papers that have been retracted, please include the rationale for doing so in the manuscript text, or remove these references and replace them with relevant current references. Any changes to the reference list should be mentioned in the rebuttal letter that accompanies your revised manuscript. If you need to cite a retracted article, indicate the article’s retracted status in the References list and also include a citation and full reference for the retraction notice.**

   We have removed several references that are no longer cited directly in the text, or are better represented by another reference already in the bibliography. Additionally, several references were added to support new text in the revised manuscript.

<u>Reviewer 1:</u>

The authors mainly consider the exposure of newly acquired N-glycosylation sites on the molecular surface as an indicator of functional relevance. However, it is also important to note that the formation of the HA trimer is essential for glycan-binding activity. Since trimerization is distinct from immune surveillance mechanisms, it may impose constraints on further molecular evolution. This aspect should be discussed in the Discussion section of the manuscript.

Several sentences have been added in the manuscript to address the trimerization of the HA.

It is generally considered that N-linked glycosylation enables viruses to evade immune surveillance mechanisms. Has the efficacy of this mechanism been evaluated using other viruses? And if there are issues to be considered in the program, what aspects should be taken into account? On this point, the authors should include their opinions in the main text, which would provide valuable information for readers conducting similar studies.

The final paragraph of the manuscript has been modified to address the first point by elaborating on the extent to which spike proteins are glycosylated in SARS-CoV-2, and the third-to-last paragraph of the manuscript has been modified to address the second point by elaborating on the weaknesses of the current approach.

<u>Reviewer 2:</u>

The program uses genetic data; however, the method by which the DNA sequence is translated into a protein sequence remains unclear. There are six open reading frames, and it is uncertain how the program will determine the correct open reading frame.

The following sentences have been added to the manuscript: “Sequences were curated to ensure they began with the correct start codon, eliminating reading frame ambiguity for translation. Sequences that did not begin with a start codon were eliminated.” This curation was performed by “ifvDataCleaner”, a small helper program found in the “additional_tools” folder of the program files.

The proposed program predicts the likely location for glycosylation; however, it does not guarantee that this site will indeed undergo glycosylation. While the exposure of the side chain increases chances of potential glycan attachment, other factors also influence whether glycosylation will occur or not. Can authors comment on that?

Several sentences describing other factors that contribute to sequon occupancy have been added to the discussion section of the manuscript.

Author did not consider oligomerization of protein in when calculation exposed potential glycosylation sites. Could it be a limitation of the program.

Several sentences have been added in the manuscript to address the trimerization of the HA.

The definition of a sequon should be clearly stated. Given that not all glycosylation sites conform to the Asn-X-Ser/Thr rule, the specific amino acid sequences the program looks for are required.

We added an explanation regarding definitions of a sequon.

Authors should provide access to the program if possible.

This has been done. The program is now accessible through GitHub and archived through Zenodo.

Sincerely,

Martin S. Zand, MD, PhD
Professor of Medicine
University of Rochester
